# Task shifting to non-physician clinicians for integrated management of hypertension and diabetes in rural Cameroon: a programme assessment at two years

**DOI:** 10.1186/1472-6963-10-339

**Published:** 2010-12-14

**Authors:** Niklaus D Labhardt, Jean-Richard Balo, Mama Ndam, Jean-Jacques Grimm, Engelbert Manga

**Affiliations:** 1 Swiss Tropical and Public Health Institute, Basel, Switzerland; 2 University of Basel, Basel, Switzerland; 3 Health District of Mbankomo, Ministry of Public Health of Cameroon, Mbankomo, Cameroon; 4 Health District of Mfou, Ministry of Public Health of Cameroon, Mfou, Cameroon; 5 Unit of Endocrinology, Diabetology, Metabolism and Nutrition, Hôpital du Jura, Porrentruy, Switzerland

## Abstract

**Background:**

The burden of non-communicable chronic diseases, such as hypertension and diabetes, increases in sub-Saharan Africa. However, the majority of the rural population does still not have access to adequate care. The objective of this study is to examine the effectiveness of integrating care for hypertension and type 2 diabetes by task shifting to non-physician clinician (NPC) facilities in eight rural health districts in Cameroon.

**Methods:**

Of the 75 NPC facilities in the area, 69 (87%) received basic equipment and training in hypertension and diabetes care. Effectiveness was assessed after two years on status of equipment, knowledge among trained NPCs, number of newly detected patients, retention of patients under care, treatment cost to patients and changes in blood pressure (BP) and fasting plasma glucose (FPG) among treated patients.

**Results:**

Two years into the programme, of 54 facilities (78%) available for re-assessment, all possessed a functional sphygmomanometer and stethoscope (65% at baseline); 96% stocked antihypertensive drugs (27% at baseline); 70% possessed a functional glucose meter and 72% stocked oral anti-diabetics (15% and 12% at baseline). NPCs' performance on multiple-choice questions of the knowledge-test was significantly improved. During a period of two years, trained NPCs initiated treatment for 796 patients with hypertension and/or diabetes. The retention of treated patients at one year was 18.1%. Hypertensive and diabetic patients paid a median monthly amount of 1.4 and 0.7 Euro respectively for their medication. Among hypertensive patients with ≥ 2 documented visits (n = 493), systolic BP decreased by 22.8 mmHg (95% CI: -20.6 to -24.9; p < 0.0001) and diastolic BP by 12.4 mmHg (-10.9 to -13.9; p < 0.0001). Among diabetic patients (n = 79) FPG decreased by 3.4 mmol/l (-2.3 to -4.5; p < 0.001).

**Conclusions:**

The integration of hypertension and diabetes into primary health care of NPC facilities in rural Cameroon was feasible in terms of equipment and training, accessible in terms of treatment cost and showed promising BP- and FPG-trends. However, low case-detection rates per NPC and a very high attrition among patients enrolled into care, limited the effectiveness of the programme.

## Background

The global burden of non-communicable chronic diseases (NCCD) falls disproportionally on low- and middle-income countries (LAMICs), which account for 80% of all deaths from NCCD worldwide. More than half of these deaths in LAMICs are attributable to cardiovascular disease (CVD) and diabetes (cancer and chronic obstructive pulmonary disease cause most of the rest) [1,2]. Moreover, morbidity and mortality due to CVD and diabetes are projected to grow at greatly increased rates in developing countries over the next decades [3]. Even in Sub-Saharan Africa (SSA), where communicable diseases still remain the major cause of death, rates of CVD and diabetes are increasing with the associated effects on chronic illness, mortality and disability. Already CVD is the second leading cause of death in SSA and the number one killer in adults older than 30 years, and the burden is projected to more than double between 1990 and 2020 in this region [4,5].

However, interventions to prevent premature deaths due to NCCDs in LAMICs are considered to be feasible, realistic and cost-effective [6] and the medical treatment of hypertension and diabetes in LAMICs has been advocated as a major strategy in preventing deaths due to non-communicable diseases [7,8]. To improve coverage, access and sustainability, primary health care in low resource settings need to include NCCD programmes as an integral part of the services, within the limits of available resources, and in particular nurses need to play a major role [9].

Task shifting is the name given to a process of delegation whereby tasks are moved, where appropriate, to less specialised health workers [10]. Non-physician clinicians (NPCs) are health-care providers who are not trained as physicians but who take on many of the diagnostic and therapeutic functions of medical doctors [11]. Recently, task shifting from physicians to NPCs has been a key element in successful scaling up of HIV-programmes within tight budgets in several LAMICs [12]. However, there are only a few reports from Africa, not all of the most recent date, on task shifting of the management of diabetes and hypertension in primary health care. Moreover, those studies examined the effectiveness in a very limited number of particularly chosen NPC-clinics [13-15].

The aim of this study is to test, in a real-life setting, the feasibility and effectiveness of the systematic integration of hypertension and diabetes into the primary health care package in eight rural Cameroon districts with 75 NPC-facilities.

## Methods

### Study design

The study aimed at investigating the effects of a programme that integrated the care for hypertension and type 2 diabetes (T2D) into the existing primary health care system by task shifting from physicians in the hospitals to NPCs in health centres. The programme was implemented in eight resource-limited Cameroonian districts with mostly public facilities. Apart from an initial donation of sphygmomanometers and glucose meters and a small stock of drugs, no additional private or public resources were made available to the providers to maintain the activities, no medication was provided free of charge and the focus was not only on a few high-performance clinics or individuals.

From March 2007 to January 2008 NPC facilities were equipped and trained for prevention, diagnosis and care of T2 D and hypertension. The programme run under the auspices of the Cameroon Ministry of Health with support for initial equipment and logistics from the *Cameroon-Jura-Switzerland Cooperation *and *Coopération Afrique*, a Swiss non-governmental organisation. In March 2009 the feasibility and effectiveness of this integrative approach was assessed.

### Study setting

The eight health districts involved in the study are situated in the Central Region of Cameroon. The combined population is estimated at about 400'000 habitants with about two thirds living in remote rural villages, the others in small towns. The area is part of the *Cameroon-Jura-Switzerland Cooperation *that has been supporting the districts' primary health care activities since 1992. Contrarily to other African countries, Cameroon does not have a specific curriculum for clinical officers. In the health centres nurses with varying levels of education take therefore the role of NPCs.

When the programme for hypertension and diabetes started in 2007, there were 79 peripheral clinics in the area offering nurse-led primary health care. Four of these had a physician among the staff; the remaining 75 were exclusively led by NPCs. Most (78%) of the peripheral clinics were public where consultations are usually free of charge but diagnostics and drugs for curative services have to be paid out-of-pocket. The area also has eight district hospitals and two missionary hospitals.

### Intervention

The actions undertaken in the current study and the outcomes assessed are summarised in Table 1. A preliminary survey was carried out in January 2007 to assess the availability of equipment and drugs for hypertension and diabetes in 75 (95%) of the 79 peripheral clinics. The subsequent intervention included training, equipment and supervision. Between March 2007 and January 2008 about 130 NPCs were trained during five three-day modules. The training comprised prevention, diagnosis and treatment of hypertension and T2 D. It was provided by four of the authors of this article (NDL, JRB, MN, EM) and focused mainly on protocol-driven care. Trained NPCs attended twice a year a one-day refresher course where they presented records of their newly identified patients.

**Table 1:** Key interventions of the programme, measured outcome-variables and their sources

Objective	Activity/intervention	Measured outcome	Method
Assess situation and needs	Preliminary survey of availability of equipment and drugs	Status of equipment and drugs for diabetes and hypertension in 2007	Investigators made inventories of 75 (95%) of the health centres (2007)

Provide equipment to health centres	Equipment with sphygmomanometers, stethoscopes and blood glucose meters. Basic medication added to pharmacy stocks.	Status of equipment and drugs for diabetes and hypertension in 2009	Investigators made inventories of equipped health centres after two years (2009)

Train NPCs	Initial three-day training modules; Six-monthly one-day refresher courses;	Knowledge of NPCs before, directly after and 2 years post-training	Multiple-choice tests before, directly after and at 2 years post-training (2007-2009)

Improve care for hypertensive and diabetic patients	Checks of equipment and patient-records integrated into the protocol of regular supervisions carried out by the district health committee.	Numbers of patients identified, treated and followed by trained NPCs (2007-2009) Change of BP and FPG among patients followed by trained NPCs (2007-2009)	Standardised medical record forms

NPC: non-physician clinicians; BP: blood pressure; FPG: fasting plasma glucose.

Immediately after training, participating NPC facilities were equipped with new sphygmomanometers, stethoscopes and blood glucose meters. A small initial stock of hydrochlorothiazide (HTZ), nifedipine (NFP), metformin (MF), glibenclamide (GBL) and urine-strips as well as strips for the glucose meters were added to the regular pharmacy stocks of every facility. Similar to all other drugs, supplies had to be renewed by the health centre through the official drug supply system of the district. Except for the initial stock, the programme provided no financial support for drugs or equipment. Key aspects of the programme, such as checking of the equipment and patient-records were integrated into the protocol of the regular supervisions carried out by the district health committee.

In January 2008, 69 (87%) of the 79 peripheral clinics had been fully equipped. Reasons for not equipping the 10 remaining centres were not having participated in training (5 clinics), under-performance during training (3) and severe non-functioning of the facility (2).

Before the start of the programme, its implementation as well as its assessment at two years was officially approved by the Ministry of Health, as well as by a local panel involving physicians, nurses and community-representatives of the eight districts. Administrative clearance to conduct and evaluate the programme was subsequently obtained from the Ministry of Health. For the programme itself, no additional request to an ethical committee was submitted. However, the applied treatment protocols and procedures received ethical clearance from the national ethics committee (authorisation N°059/CNE/DNM/08) as they were submitted for a trial that was nested within the programme described in this paper (NCT00744458, unpublished). At data entry, records were handled confidentially and all processed data were anonymous.

### Treatment protocols

Treatment protocols were based on international recommendations [16-19] and adapted to the local conditions, considering safety, availability and prices of drugs.

Blood pressure had to be measured manually using new sphygmomanometers. Four measurements ≥ 140/90 mmHg after 15 minutes rest at two different days were required before starting treatment. Depending on the WHO grade of hypertension and the individual risk profile, medical treatment was either started immediately or an initial trial with changes in lifestyle and diet was done. First line treatment was low-dose HCTZ (12.5 or 25 mg), to be combined with NFP if necessary. In patients with diabetes or impaired fasting glucose, NFP replaced HCTZ as a first-line drug. If combination therapy was unsuccessful, the guidelines recommended referral to a physician.

Blood glucose measurements had to be done using new glucose meters that calculate the plasma equivalent of the whole blood glucose measurement. Two fasting blood glucose levels ≥ 7 mmol/l were required for diagnosis before starting treatment. Depending on the level of fasting plasma glucose (FPG), medical treatment was either started immediately or delayed while changes in lifestyle and dietary habits were prescribed. If there was no contra-indication, MF was used as a first-line therapy. If monotherapy was unsuccessful or there were contraindications to MF, GBL could be introduced. Patients presenting with pre-coma or coma, detectable ketone bodies in the urine and patients not responding to dual therapy, had to be referred to a physician. Management of T2 D patients requiring insulin remained the responsibility of physicians at a hospital level. All drugs used are part of the Cameroonian Essential Drug List, available over the national drug distribution system and are sold to patients at a relatively low cost: HCTZ 50 mg (10 CFA), NFP 10 mg (10 CFA), MF 500 mg (15 CFA), GBL 5 mg (10 CFA); FPG-measurement was taxed at 800 CFA and ketone-bodies in the urine at 200 CFA; 650 CFA equal 1 Euro.

### Measured outcome variables and data-collection

Data presented in this paper originate from two points in time: the start of the programme and the assessment after two years. At the beginning, investigators visited NPC-facilities for a baseline survey to check the availability of equipment and drugs essential to care for diabetes and hypertension. NPCs were invited to participate in a knowledge assessment by a multiple-choice (MC) questionnaire before and - in different order and layout-directly after the training (pre- and post-test).

Two years later, all 69 health centres that participated in the programme were invited for assessment. At that moment, the following four outcome variables were measured (see Table 1): Status of equipment in formerly equipped NPC-facilities; status of knowledge among trained NPCs; number of patients identified and followed by trained NPCs; and the trend of BP and FPG in patients treated by trained NPCs. To assess the status of equipment, the same checklist was used as for the inventory in the baseline survey. Status of knowledge was assessed with the same MC-questions as at baseline but in different order and layout. Data on patients, the prescribed treatment and BP/FPG trends were obtained from standardised medical record forms, provided to NPCs immediately after the training. These covered a minimum of information the NPC in charge had to enter on hypertensive or diabetic patients on every consultation (BP, FPG, abdominal circumference, weight, height and prescribed treatment). We defined abdominal obesity as a waist circumference of ≥ 80 cm for women and ≥ 94 cm for men, a definition already used for different surveys conducted in Cameroon [20]. Loss to follow-up was defined as no recorded consultation for at least three months. All patient-records available were entered into EpiData^® ^3.1 by two different persons using double entry mode in order to ensure accuracy of processed data.

### Statistical analysis

T-test and Chi2-test were used as appropriate. The performance of nurses in the knowledge MC at two years was assessed using a linear regression with the nurses' sex, age, level of education and professional experience being covariates. To identify reasons for lost to follow-up, we used a cox-regression model including only those patients who should have completed at least one year of follow-up at the time of data-collection. Its co-variates were the patients' sex, age, WHO-grade of hypertension (1,2,3), co-morbidity with diabetes and the type of institution (missionary versus public). Treatment response of patients was assessed as the change in BP- or FPG-values between the visit where treatment was started and the last recorded consultation. For analysis of this outcome, patients not completing at least one follow-up visit and patients with missing data during follow-up were excluded. Univariate and multiple linear regressions were used to estimate the crude and adjusted delta in BP and FPG. In case of hypertension, adjustment was done for the patients' sex, age, abdominal obesity and co-morbidity with diabetes or impaired fasting glucose. In case of diabetes, the changes in FPG were adjusted for sex, age, abdominal obesity and co-morbidity with hypertension. A second multiple linear regression model was run for BP- and FPG-changes that includes in a addition to the first model the height of BP (WHO-grade) or FPG (< 11 mmol/l versus ≥ 11 mmol/l) at the start of the treatment, the number of consecutive follow-up visits and the variable pharmacological versus non-pharmacological treatment. Intracluster correlation was assessed for BP- and FPG-trends, using generalized linear mixed models, but was found to be small (ICC < 0.05). Therefore accounting for multi-centre clustering has been dropped from analysis. All reported confidence intervals are 95% intervals. Statistical analysis was performed on STATA^® ^10.1.

## Results

### Baseline

Among the 75 of the peripheral clinics that were assessed in the preliminary survey, 49 (65%) disposed of a well functioning sphygmomanometer and stethoscope and 11 (15%) had a functional glucose-meter. Twenty (27%) had at least one anti-hypertensive drug on stock and nine (12%) at least one oral anti-diabetic. According to the head-nurses of the facilities, 39 (52%) and 65 (87%) centres had not diagnosed, referred or treated a single patient with high BP or diabetes, respectively, over the last six months. The others stated to follow a median number of 3 (interquartile range: 2-6) hypertensive and 1 (1-2) diabetic patients. Drugs used to treat hypertension and diabetes varied between facilities. For hypertension it was mainly furosemide, alpha-methyl dopa or diazepam; for diabetes sulphonylureas or metformin were used. Missionary clinics were more likely to dispose of equipment and drugs (p < 0.01) and followed on average more patients with either diabetes or hypertension (p < 0.01).

### Participation

At two years, the status of equipment was assessed in 54 (78%) of the 69 formerly equipped facilities. Fifteen centres were unavailable for the following reasons: complete change of staff without successful handing over of activities related to hypertension and diabetes (9 centres), transitional or permanent close-down of the facility (4) not reachable (2). Of the 54 clinics, 46 (85%) were public and eight (15%) were missionary health centres.

A total of 62 (48%) of the initially 130 trained nurses participated in the knowledge-assessment at two years. Reasons for non-participation of trained nurses in the assessment were reallocation to other districts (42 nurses) and others (24). Reallocation of staff only concerned the public facilities. The characteristics of the assessed nurses are displayed in Table 2.

**Table 2:** Characteristics of the 62 non-physician clinicians participating in the knowledge assessment at two years.

**Age (standard deviation)**	44 (8)
**Sex**	Female 26%
**Level of medical education**	Nurse assistant 13%
	Staff nurse 31%
	Registered nurse 56%
**Years of professional experience (SD)**	18 (9)
**Years working at the current institution (SD)**	6 (5)

A total of 804 patient records were collected from 81 nurses of 57 health centres and 796 (99%) were analyzed; the remaining eight had missing or non-readable BP- or FPG-values.

### Outcomes

#### Status of equipment in the facilities

Compared to baseline, the status of equipment was highly improved after two years (see Table 3). Reasons of 16 glucose meters being rated as non-functional at two years were stock-out of compatible strips or batteries (15 units) and robbery (1).

**Table 3:** Inventory of equipment and drugs in the 54 health centres that participated in both assessments.

	Preliminary survey	2-year assessment
**Equipped to measure BP**	67%	100%
**Functional glucose meter**	14%	70%
**At least one anti-hypertensive drug**	20%	96%
**A least one oral anti-diabetic drug**	12%	72%

Equipped to measure BP was defined as having a sphygmomanometer and a stethoscope without leakages or any other visible damages. A functional glucose meter meant that the battery was working and that compatible strips were available. BP: blood pressure.

#### NPC state of knowledge

MC-test results before and directly after training were available from 70 nurses and from all the 62 nurses participating in the two-year assessment. Of these, 35% had already participated in two, 55% in one and the remaining 10% in none of the refresher courses. Knowledge about hypertension and diabetes among nurses was poor before training, increased directly after training and remained high at the two-year assessment (see Table 4). In multiple linear regression analysis, the overall test-performance at two years was independent of the nurses' sex, age, medical education and professional experience.

**Table 4:** Percentage of non-physician clinicians choosing the correct answer from five alternatives in the multiple-choice (MC) questions.

MC-question	Pre-test (n = 70)	Post-test (n = 70)	2-year-test (n = 62)
**Knows that diabetes is diagnosed based on FPG**	93%	100%	94%

**Knows BP levels defining arterial hypertension**	20%	80%	69%

**Chooses a correct drug for first line treatment of hypertension**	17%	94%	95%

**Chooses an antihypertensive drug that is not contra-indicated for treatment during pregnancy**	27%	97%	82%

**Chooses lifestyle and diet modifications for the treatment of IFG**	73%	97%	97%

**Chooses the correct protocol for moderate diabetes (FPG 7-10.9 mmol/l)**	30%	44%	47%

**Chooses the correct protocol for severe diabetes (FPG > 11 mmol/l)**	23%	76%	79%

**Chooses to search for ketone bodies in the urine in case of very high blood glucose levels**	41%	96%	95%

**Chooses the right patients who must be referred immediately to a hospital (coma and ketoacidosis)**	46%	54%	58%

BP: blood pressure; FPG: fasting plasma glucose

### Recruited Patients

The number of recruited patients per trained NPC varied from one to 51. Table 5 displays characteristics of the 796 patients of whom data were analyzed. Forty-one (28%) of the diabetic and 192 (27%) of hypertensive patients were aware of their condition and declared having once received treatment in another facility, but none was under medical treatment at the moment they were diagnosed by the NPCs. In the 54 facilities where pre- and post-intervention data are available, the median number of patients per facility with hypertension increased from 0 (interquartile range 0-2) to 14.5 (7-22) and from 0 (0-0) to 3 (1-6) for diabetes.

**Table 5:** Characteristics of the 796 newly recruited patients in 57 NPC facilities.

Patient characteristics	
**Sex**	Female 69%
**Age in years (standard deviation)**	60 (12)
**Abdominal Obesity**	52.3%
**Patients with hypertension (n = 732)**	
- **WHO Grade 1 (140-159/90-99 mmHg)**	25.2%
- **WHO Grade 2 (160-179/100-109 mmHg)**	35.3%
- **WHO Grade 3 (≥ 180/110 mmHg)**	39.5%
- **Hypertension and IFG**	18.7%
**Patients with diabetes (n = 144)**	
- **FPG 7-10.9 mmol/l**	52.8%
- **FPG ≥ 11 mmol/l**	47.2%
- **Co-morbidity with diabetes and hypertension**	61.8%

Abdominal obesity was defined as a waist circumference ≥ 80 cm for women and ≥ 94 cm for men. FPG: Fasting plasma glucose; IFG: Impaired fasting glucose (5.6-6.9 mmol/l).

**Figure 1:**
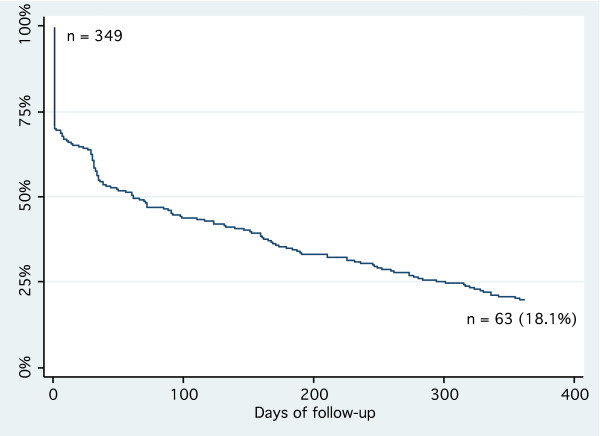
**One-year retention of diabetic and hypertensive patients**. Attrition among the 349 patients who were recruited at least 15 months before the assessment. Lost to follow-up was defined as no recorded consultation for ≥ 3 months.

A total of 2579 visits and a follow-up time of 222 patient-years were recorded. Of the 796 patients, 562 (70.6%) were lost to follow up at the time data were collected (no recorded visit since ≥ 3 months). Before dropping out, these patients completed a median number of 2 visits (interquartile range: 1-3) and a median follow-up time of 28 days (0-92). Among the patients who were recruited at least 15 months before the assessment (n = 349), only 18.1% were still retained in the programme at one year (see Figure 1). In a cox regression model none of the assessed patient-characteristics was associated with the risk of being lost to follow-up at one year. However, patients of missionary clinics were at higher risk of being lost to follow-up (see Table 6).

**Table 6:** Assessed patient-characteristics and their association to the risk of being lost to follow-up at one year.

Independent variable	Hazard-ratio (95% confidence interval)	p-value
**Sex**	1.08 (0.82 to 1.41)	0.590
**Age**	1.00 (0.99 to 1.01)	0.653
**Diabetes**	0.81 (0.56 to 1.17)	0.262
**WHO-grade of hypertension**	0.93 (0.80 to 1.07)	0.315
**Missionary facility**	1.74 (1.19 to 2.53)	0.004

Hazard-ratios and p-values are derived from a cox regression with lost to follow-up at one year being the dependent variable.

### Prescribed treatment and BP-changes in hypertensive patients

All 732 patients with hypertension received counselling on diet and lifestyle. NPCs prescribed at the first visit hydrochlorothiazide monotherapy to 317 (43.3%), nifedipine monotherapy to 114 (15.6%) and bitherapy to 120 (16.4%) patients. The remaining 181 (24.7%) were on diet and lifestyle change alone. At the last visit recorded, the NPC prescribed to 272 (37.2%) hydrochlorothiazide monotherapy, to 103 (14.1%) nifedipine monotherapy and to 236 (32.2%) a bitherapy. The remaining 121 (16.5%) were still on non-pharmacological treatment alone. Patients on medication paid a median monthly amount of 1.4 Euro (interquartile range 1.2-1.5) for the prescribed anti-hypertensive drugs.

**Figure 2:**
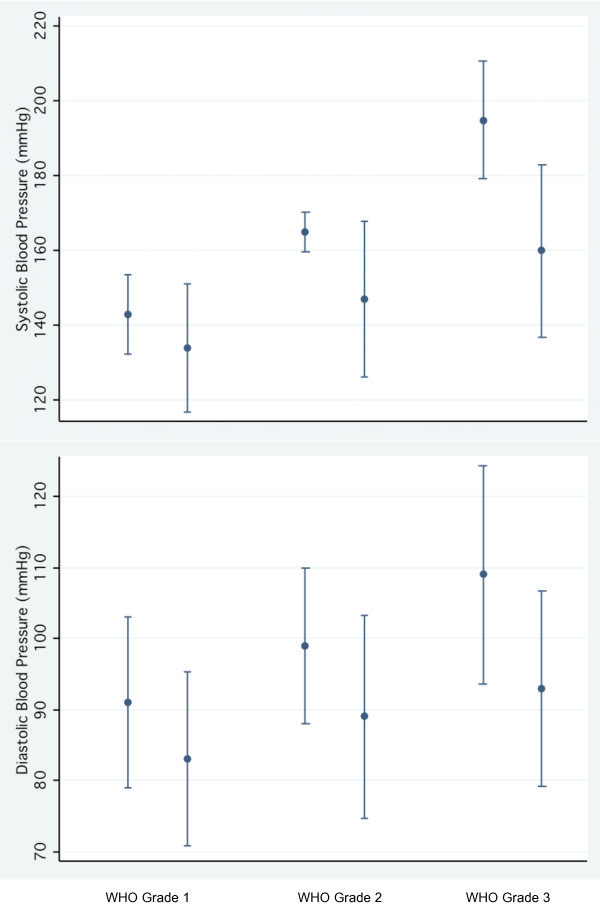
**Change of blood pressure among hypertensive patients**. Changes in systolic and diastolic blood pressure between the visit where treatment was started (usually the second visit) and the last follow-up visit. Dots are displaying mean values, bars the 95% confidence interval. Groups are according to the WHO grade of hypertension at initiation of therapy. Grade 1 n = 82; grade 2 n = 160; grade 3 n = 196). All changes are significant (p < 0.001).

The 493 hypertensive patients with at least one documented follow-up visit were included in analysis of BP-change. This cohort has a median follow-up of 102 (35-240) days with a median of 3 (2-6) documented visits. Fifty-five patients (11%) in the cohort were still on non-pharmacological treatment alone at the last visit recorded. Unadjusted overall systolic BP decreased by 22.8 mmHg (95% CI: -20.6 to -24.9) and diastolic BP by 12.4 mmHg (95%CI: -10.9 to -13.9). Figure 2 displays the BP at baseline (initiation of treatment) and at the last consultation by WHO grade of hypertension at the first visit. Adjusted for patient-characteristics (sex, age, abdominal obesity, co-morbidity with diabetes or impaired fasting glucose) the BP-decrease remained significant: -26.5 mmHg (95%CI: -12.5 to -40.5) for systolic and -17.2 mmHg (95%CI: -7.1 to -27.3) for diastolic BP.

In the second model of multiple linear regression analysis, higher initial WHO-grade of hypertension (coefficients: grade 1 to grade 2: -9.2 (CI95%: -3.7 to -14.8); grade 1 to 3: -27.3 (95%CI: -21.7 to -32.9)) and a greater number of consecutive consultations (coefficient: -1.3; 95%CI: -0.6 to -1.9) were significantly associated with a greater decrease in systolic BP. The patients' sex, age, co-morbidity with diabetes, abdominal obesity, impaired fasting glucose and pharmacological treatment were not significantly related. The same model for the diastolic BP-trend showed a similar result with WHO-grade (coefficients: grade 1 to 2: -4.5 (-0.1 to -8.9); grade 1 to 3: -11.3 (95%CI: -6.9 to -15.7)) and the number of consultations (coefficient: -0.9 (-0.3 to -1.5)) being the only two covariates significantly associated with diastolic BP-decrease.

### Prescribed treatment and FPG-change in diabetic patients

All of the 144 patients with diabetes received counselling on diet and lifestyle. At the first visit, NPCs prescribed to 67 (46.5%) metformin and to 2 (1.4%) glibenclamide. At the last consultation recorded, the NPCs prescribed to 72 (50%) metformin monotherapy, to 2 (1.4%) glibenclamide monotherapy and to 9 (6.3%) bitherapy. The remaining patients were still on diet and lifestyle change alone. Patients on medication paid a median monthly amount of 0.7 Euro (interquartile range: 0.7-2.1) for the prescribed anti-diabetic drugs.

**Figure 3:**
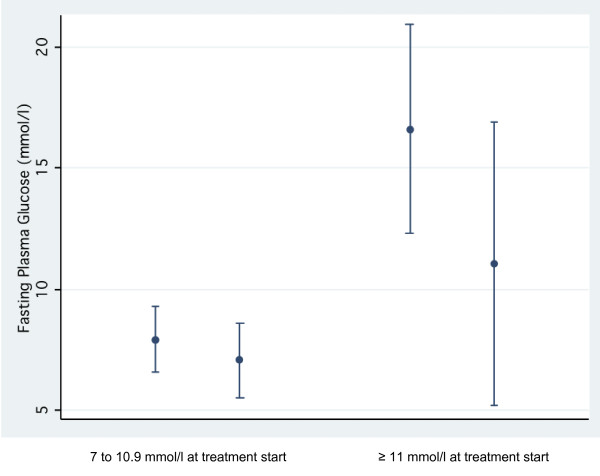
**Change of fasting plasma glucose among diabetic patients**. Change in fasting plasma glucose levels between the visit where treatment was started (usually the second visit) and the last follow-up visit among diabetic patients. Dots are displaying mean-values, with brackets indicating the 95% confidence interval. The changes in both groups are significant (sample with 7-10.9 mmol/l at the start of the treatment (n = 35), p-value = 0.0151; sample with≥ 11 mmol/l at the start of the treatment (n = 44), p < 0.001).

Of the total of 144 diabetic patients 94 (65%) completed at least one follow-up visit. Only the 79 (55%) patients where FPG-values at baseline as well as at the last consultation are available were included in the analysis of FPG-change. This cohort has a median follow-up time of 72 (23-243) days with a median of 3 (2-6) documented consultations; 15 (19%) were still on non-pharmacological treatment at the last consultation recorded. Unadjusted overall FPG decreased by 3.4 mmol/l (95% CI: -2.3 to -4.5; p < 0.001). Adjusted for patient-characteristics (sex, age, abdominal obesity, co-morbidity with hypertension) the FPG-decrease remained significant at -7.8 mmol/l (95%CI: -1.8 to -13.8). As shown in Figure 3, those with a higher initial FPG showed a steeper decrease.

In the second model of multiple linear regression analysis, high initial FPG-levels (coefficient < 11 mmol/l versus ≥ 11 mmol/l: -3.8 (95%CI: -1.2 to -6.4)) and a higher number of consultations (coefficient: -0.4 (95%CI: -0.1 to -0.7)) were significantly associated with a greater decrease of blood glucose levels. The other covariates (sex, age, co-morbidity with hypertension, abdominal obesity and pharmacological treatment) showed no significant relation with FPG-changes.

## Discussion

Cameroon, like most of SSA, faces a looming epidemic of arterial hypertension and diabetes which will put an immense strain on the general primary health care system over the years to come [21,22] but for which so far very few resources have been dedicated by the WHO and other big international donors [23]. The country is taking measures to combat the raising numbers of cases: Jean-Claude Mbanya, president of the International diabetes Federation, initiated the Cameroon Burden of Diabetes project (CAMBoD), which conducted important baseline surveys, numerous awareness activities and installed pilot clinics. This paved the way to a national programme in 2004 that aims to promote equitable access to quality health services in order to reduce the morbidity and mortality linked to diabetes and hypertension [24]. However, a majority of people with hypertension or diabetes in Cameroon are still not aware of their condition and do not have access to qualified care [25,26].

Our study highlights some of the enormous difficulties, not all of economic nature, faced by primary health care programmes for chronic diseases in SSA countries. As the preliminary survey conducted at the start of our research showed, the majority of health centres in rural Cameroon currently neither dispose of adequate equipment and drugs, nor do caregivers possess the necessary knowledge to recognise, diagnose and treat hypertension and diabetes. The experiences from our programme indicate that task shifting on a primary health care level is indeed feasible within the available resources in Cameroon: NPCs were trained and equipped with relatively few efforts to efficiently care for patients with hypertension and T2 D. But despite significantly improved clinical outcomes on BP and FPG, the overall effectiveness of the programme was modest because of limited access to patients and a very high drop out rate.

There are four major limitations to be considered in the interpretation of the reported BP- and FPG-changes: first, we describe BP- and FPG-trends originating from routinely filled in medical record forms; second, baseline BP- and FPG-values originate from the measurement when treatment was started (usually at the second visit). Studies have shown, that measurements at the first or second visit may overestimate the degree of arterial hypertension [27]. Third, our cohort cannot be compared to a control group; Fourth, the overall time of follow-up is short. The very positive trend in BP- and FPG-values in this cohort has therefore to be interpreted with caution as it may partly be due to a natural regression to the mean.

From an operational point of view, there are another three very important limitations in the assessment of the programme. First, only data from 78% of formerly equipped facilities and 48% of formerly trained NPCs were available for analysis. Most of the drop-out of centres and NPCs were due to changes of staff and/or transfer to new facilities. It is part of the national health policy to move the staff in public health care facilities regularly. Experience from our programme has shown, that after change among the staff in a facility, the program often becomes un-functional. Constant training of staff newly allocated to the facilities has therefore to be done. This can currently only be done with support from external donors and poses a big challenge to sustainability. To strengthen the teaching about care for hypertension and diabetes at a national level during the basic training curriculum of nurses and to extend the programme on a national level may on the long run contribute to sustainability. Second, the nurses' knowledge was only assessed through multiple-choice questionnaires and does not necessarily reflect their medical practice. However, an independent observational assessment of practice would have been too resource-intensive to our study. Third, we do not have any information about what happened to the patients lost to follow-up. Indeed, in regression analysis basic patient-characteristics were not related to the risk of being lost to follow-up. However, to interpret these findings, more data about the patients, such as their socio-economic status or their distance to the facility, is needed. Moreover, a subsequent retracing with an assessment of their outcomes and the reasons for attrition would be of great importance to improve the programme.

Several recent studies show that integrated nurse-led care for HIV/AIDS can be effective even on a large scale [12] and evidence is growing that task shifting may also work for other chronic conditions, such as epilepsy [28]. It is unclear how far these promising results can be generalised to asymptomatic chronic diseases, but it is clear that some problems will be the same, most importantly perhaps low retention rates [29]. Similar to our work, studies on hypertension and diabetes programmes from Dar es Saalam and Soweto respectively report very low adherence and retention rates as main challenges [30,31]. Two papers that were recently published, describe a cohort of diabetic and hypertensive patients followed between 1998 and 2000 in five NPC-facilities in Cameroon [14,15]. Similarly to our study, they report high attrition rates, especially during the first three visits. Patients from rural clinics and patients who were recruited through particular awareness- and screening-activities were more likely to remain retained. Awah et al. report from Cameroon, that culturally affected illness and treatment perceptions play a major role in attrition and adherence of diabetic patients. While modern medicine only provides care and control for diabetes, many patients seem to seek definite cure in traditional medicine [32]. However, one must assume that a high attrition rate will remain a main challenge to the effectiveness of programmes for chronic conditions on a larger scale in SSA. Further research on how to improve retention is urgently needed.

We are not aware of any publication that tested task shifting on a larger scale within the framework of existing resources for hypertension and diabetes in low-income African countries. Kengne et al. demonstrate a successful task shifting for diabetes and hypertension in three urban and two rural clinics in Cameroon. However, the experience is limited on five clinics and a high proportion of included patients were urban [14,15]. In contrast, we examined a district-wide integration without additional resources into existing rural NPC clinics that have originally been set up to care for acute infectious diseases, a situation mirroring the current reality in the field.

While acknowledging the high importance of low retention rates, our data still show, that such an integrative approach is a feasible option in settings with low resources to respond to the need for accessible care among the raising number of individuals with hypertension and diabetes. Hypertensive and diabetic patients paid for their medication a median monthly amount of 1.4 and 0.7 Euro respectively. Indeed, these amounts may still be too high for some patients in a country where poverty is wide spread. However, we can assume that for a high number of hypertensive and diabetic individuals in the study area, treatment has now become affordable. Most patients reached by our programme were unaware of their condition even though a considerable number had severe hypertension (grade 3) or diabetes with high FPG levels. In the majority of these participating patients, BP and/or FPG were reduced sharply during follow-up. Thus the potential public-benefits even of modestly successful programmes may be substantial.

However, if policy makers aim at task-shifting as an integrative approach for NCCDs on a large scale, problems encountered in this study should be taken into account: 1, Frequent reallocations of trained staff outside the study area with subsequent cessation of the activities related to diabetes and hypertension in some of these facilities; 2, Interruptions in the supply of oral anti-diabetics and strips for glucose meters; 3, low detection rates per trained and equipped NPC; and 4, a very high proportion of loss to follow-up among treated patients. While the first two points may be a problem of management, the issue of detection and attrition point to a general problem of access to care and retention in many primary health care systems in SSA. Integrated programmes for chronic diseases might provide a good opportunity to identify these problems and to take measures to strengthen the health system in general.

Further research in this field should include the following topics: comparison of patient outcomes between nurse-led and physician-led care, examination of the reasons for lost to follow-up, assessment of drug-adherence, trials testing simple interventions to improve retention and a long time cohort study with sufficient power to measure harder outcomes (i.e. stroke incidence, amputation-rate, mortality, etc.).

## Conclusions

The programme aimed at integrating the care for hypertension and diabetes into the daily routine of nurse-led primary health care in rural Cameroon. It worked within the given framework of the national public health system and except for training and initial equipment no additional resources were allocated. The presented data of the two-year assessment suggest that such an approach is feasible and that trained NPCs may treat these two conditions successfully, using affordable drugs from the national essential drug list. However, the average case-detection rate per trained NPC was low and a very high attrition among treated patients poses the main challenge.

## Competing interests

The authors declare that they have no competing interests.

## Authors' contributions

NDL headed the conceptualisation of the programme, organised the data collection, performed the statistical analysis and wrote the manuscript. JRB and MN were involved in the conceptualisation of the programme, the data collection and commented the manuscript. JJG was involved in the conceptualisation of the programme and reviewed the draft of the manuscript. EM headed the implementation of the programme, supervised the data collection and commented the manuscript. All authors red and approved the final version of the manuscript.

## Pre-publication history

The pre-publication history for this paper can be accessed here:

http://www.biomedcentral.com/1472-6963/10/339/prepub
